# Role of SIRT7 in Prostate Cancer Progression: New Insight Into Potential Therapeutic Target

**DOI:** 10.1002/cam4.70786

**Published:** 2025-04-01

**Authors:** Jiale Zhang, Chenxin Liu, Wenting Luo, Baoqing Sun

**Affiliations:** ^1^ Department of Clinical Laboratory, National Center for Respiratory Medicine, National Clinical Research Center for Respiratory Disease, State Key Laboratory of Respiratory Disease Guangzhou Institute of Respiratory Health, the First Affiliated Hospital of Guangzhou Medical University Guangzhou Guangdong China; ^2^ Guangzhou Laboratory Guangzhou China

**Keywords:** deacetylation, histone modification, prostate cancer (PCa), Sirtuin 7 (SIRT7), tumorigenesis

## Abstract

Prostate cancer (PCa) is the second most common cancer in men worldwide, and understanding its molecular mechanisms is crucial for developing effective treatment strategies. SIRT7, a NAD+‐dependent histone deacetylase, has emerged as a key regulator in PCa progression due to its roles in chromatin remodeling, DNA repair, and transcriptional regulation. Analysis of 492 PCa samples from The Cancer Genome Atlas (TCGA) via cBioPortal revealed that high SIRT7 expression is associated with poor prognosis in PCa patients. Mechanistically, SIRT7 deacetylates histone H3 at lysine 18 (H3K18Ac), a marker associated with aggressive tumors, suppressing tumor suppressor genes and promoting cancer cell proliferation and survival. Epithelial‐mesenchymal transition (EMT) is a cellular biological process in which epithelial cells undergo specific molecular and morphological changes to transform into cells with characteristics of mesenchymal cells. SIRT7 also regulates EMT, and inhibiting SIRT7 in PCa cell lines reduces cell migration and invasion, highlighting its potential as a therapeutic target. In summary, the clinical significance of SIRT7 expression in PCa requires further research to elucidate its mechanisms. Developing specific inhibitors targeting SIRT7's deacetylase activity is a promising therapeutic strategy. SIRT7 plays a crucial role in regulating biological processes such as cell proliferation, cell cycle, and apoptosis in PCa through its epigenetic control of gene expression and maintenance of genomic stability. Therefore, SIRT7 may be a potential therapeutic target for PCa, and its expression could have prognostic value for PCa patients, providing important guidance for clinical monitoring and diagnosis by physicians.

## 1 Introduction

Sirtuins are a highly conserved family of proteins found across all domains of life, from archaea to humans. They were first discovered in budding yeast [1, 2]. The Sirtuin protein family consists of a class of nicotinamide adenine dinucleotide (NAD+)‐dependent histone deacetylases (HDACs), classified as Class III HDACs, and is homologous to the yeast silent information regulator 2 (Sir2) [3]. In mammals, seven Sir2‐like enzymes (SIRT1‐7) have been identified, all of which possess highly conserved NAD+ and catalytic domains [4]. These enzymes exhibit diverse subcellular localizations, enzymatic activities, molecular targets, and biological functions (Table 1). Sirtuins play crucial regulatory roles by interacting with various substrates, including maintaining genomic stability [58], regulating cellular senescence [59, 60, 61], modulating metabolism [2], facilitating ribosome biogenesis [62, 63], and influencing tumor biology.

**TABLE 1:** Biological overview of the sirtuin protein family in mammals (adapted and updated from Poulose et al. [5] and Vincenzo et al. [6]).

Sirtuins	Subcellular localization	Enzymatic activity	Histone targets	Biological functions	Related to tumors
SIRT1	Nucleus [7], cytoplasm [8]	Deacetylase	H1‐K26Ac H3‐K9Ac H4‐K16Ac H4‐K20me H3‐K9me3	Metabolism [9, 10], cell cycle [11], apoptosis [7, 12], cell migration [13], tumorigenesis [14, 15], tumor suppression [16], lifespan [17], circadian rhythm control [18, 19], insulin signaling [20]	Prostate cancer, breast cancer, lung cancer, colon cancer, ovarian cancer, gastric cancer, lymphoblastic leukemia, chronic lymphocytic leukemia, diffuse large B‐cell lymphoma, chronic myeloid leukemia, melanoma, glioma, bladder cancer
SIRT2	Cytoplasm [21], Nucleus [22]	Deacetylase	H3‐K18Ac, H3‐K56Ac, H4‐K16Ac	Cell cycle [23], genomic stability [23], longevity [24], autophagy [25], immune response [26]	Leukemia, neuroblastoma, hepatocellular carcinoma, pancreatic cancer, breast glioma, head and neck squamous cell carcinoma, breast cancer, prostate cancer, esophageal cancer, ovarian cancer, glioma
SIRT3	Mitochondria [27]	Deacetylase		TCA cycle [28], urea cycle [29], fatty acid oxidation [29, 30], oxidative phosphorylation [31], longevity [32]	Colon cancer, gastric cancer, oral squamous cell carcinoma, esophageal cancer, kidney cancer, bladder cancer, melanoma, breast cancer, leukemia (B‐cell chronic lymphocytic leukemia, mantle cell lymphoma, chronic lymphocytic leukemia)
SIRT4	Mitochondria [33]	ADP‐Ribosyltransferase		TCA Cycle [34], Insulin Secretion [34, 35], Fatty Acid Oxidation [36], Tumor Suppression [37]	colorectal cancer, burkitt lymphoma, bladder cancer, breast cancer, colon cancer, ovarian cancer, thyroid cancer
SIRT5	Mitochondria [33]	Deacetylase, desuccinylase, demalonylase, deglutarylase		Urea cycle [38], fatty acid oxidation [39], ketone body synthesis [40], Oxidative Stress [41, 42]	Nonsmall cell lung cancer, colorectal lung cancer, pancreatic cancer, breast cancer
SIRT6	Nucleus [43]	Deacetylase, ADP‐Ribosyltransferase, demyristoylase, depalmitoylase	H3‐K9Ac, H3‐K56Ac, H3‐K18Ac	Genomic stability [44], protein secretion [45], lifespan [46]	Lung cancer, prostate cancer, melanoma, nonmelanoma skin cancer, pancreatic cancer, breast cancer, colon cancer, hepatocellular carcinoma
SIRT7	Nucleus [33], cytoplasm [47]	Deacetylase, desuccinylase	H3‐K18Ac	Transcription control [48], genomic stability [49], aging [50, 51, 52], stress response [53, 54, 55, 56], tumorigenesis [48, 57]	Breast cancer, colorectal cancer, hepatocellular carcinoma, osteosarcoma, ovarian cancer, prostate cancer, thyroid cancer

Although the research on SIRT7 within the sirtuin family is not yet fully elucidated, it participates in various vital physiological processes, including rRNA synthesis, ribosome biogenesis, chromatin remodeling [64], DNA repair [65], and transcriptional regulation. Moreover, increasing evidence suggests that SIRT7 acts as a key transcription factor in the development and progression of several cancers by regulating epigenetics [66], maintaining malignant cell phenotypes [48], and controlling tumor‐specific growth [67], invasion, and metastasis [68]. The expression levels of the SIRT7 gene vary across different human organs and tissues, where it can function as either a positive or negative regulatory factor.

Our previous analysis using the cBioPortal for Cancer Genomics (www.cbioportal.org) examined transcriptomic and copy number variation data from 492 prostate adenocarcinoma samples in The Cancer Genome Atlas (TCGA). We found that high SIRT7 expression is correlated with poor prognosis in prostate cancer (PCa) patients. However, the mechanistic roles and clinical implications of SIRT7 expression in tumor development and progression remain unclear. Further in‐depth studies are required to elucidate its functional mechanisms and potential as a therapeutic target in human PCa. There is also experimental evidence showing that SIRT7 primarily promotes PCa progression by upregulating the androgen receptor (AR) signaling pathway, thereby enhancing cell proliferation, and androgen‐induced autophagy, and aggressiveness [69].

## 2 Gene Structure and Subcellular Localization of SIRT7

SIRT7 is a class III histone deacetylase that belongs to the sirtuin family [70]. Plays a crucial role in cellular signal transduction and various biological processes [71]. The SIRT7 gene is located on the long arm of chromosome 17 at band 25.3 (17q25.3). It spans 6.2 kb of genomic DNA and consists of 10 exons, with exons 3–9 encoding the deacetylase catalytic domain of the SIRT7 protein, comprising 275 amino acids [72, 73] SIRT7 is a single‐copy gene, and its protein product consists of 400 amino acids, with a molecular weight of approximately 44.9 kDa. Both the N‐terminal and C‐terminal regions can bind different substrates. SIRT6 and SIRT7 are predominantly found in the nucleus [74]. The nuclear localization signal (NLS) within the SIRT7 protein sequence is identified as 61LQGRSRRREGLKRRQE76, and the nucleolus localization signals (NoLS) are 392KRTKRKKVT400. This unique localization links SIRT7 to rDNA transcription, ribosome biogenesis, and cell proliferation. There are studies suggesting that SIRT7 is involved in regulating cardiac stress response and cell apoptosis [75]. Moreover, studies have found that SIRT7 is widely expressed in various proliferative cancers [64]. Its expression is upregulated in several tumor types, including PCa, ovarian cancer (OC), liver hepatocellular carcinoma (LIHC), rectum adenocarcinoma (READ), breast invasive carcinoma (BRAD), and thyroid carcinoma (THCA) [57, 76, 77, 78, 79, 80]. The high expression levels of SIRT7 are consistent with the characteristics of high synthesis and high metabolism observed in tumor cells.

## 3 Biological Functions of SIRT7

### 3.1 Deacetylase Activity

Histone deacetylases (HDACs) are a class of proteases associated with epigenetic regulation, playing crucial roles in chromatin structure modification and gene expression regulation. SIRT7 is an NAD(+)‐dependent H3K18Ac (acetylated lysine 18 of histone H3) deacetylase that stabilizes the transformed state of cancer cells [75]. Studies have shown that the acetylated lysine residue 18 on histone H3 (H3K18Ac) is predominantly found at transcription start sites (TSS) of genes [81]. SIRT7 exhibits high selectivity for H3K18Ac and can bind to the promoters of specific gene targets, exerting transcriptional repression. Under normal conditions, the acetylation level in the nucleus is dynamically regulated by the activities of histone acetyltransferases (HATs) and HDACs, maintaining a balanced state [82, 83]. SIRT7 is a site‐specific enzyme whose deacetylation activity for H3K18 is limited to certain gene promoters and does not completely alter the acetylation level of H3K18. HDACs, including SIRT7, are typically overexpressed in cancer cells, enhancing deacetylation, leading to tighter binding of histones to DNA, which is unfavorable for gene transcription. SIRT7, in conjunction with the transcription factor ELK4, participates in downregulating tumor suppressor genes. Overexpression of ELK4 can increase SIRT7 levels at specific TSG loci, maintaining low acetylation levels of H3K18 at multiple TSG promoters, thus contributing to tumorigenesis and maintaining the malignant phenotype of cancer cells, correlating with poor patient prognosis [84]. Consequently, SIRT7 is considered a crucial potential target for drug design against various diseases.

### 3.2 Maintenance of Genomic Stability

Loss of genome‐wide epigenetic stability is a common feature of many cancers. The declining DNA repair capacity with age leads to damage accumulation, which can induce genetic mutations and ultimately cancer. Sirtuins regulate DNA repair processes [70, 85]; nonhomologous end joining (NHEJ) is a repair mechanism for DNA double‐strand breaks. It is one of the most important DNA repair pathways in cells, especially during the G1 phase of the cell cycle, when homologous recombination cannot proceed. In such cases, NHEJ is the primary repair pathway. SIRT7 plays a role in DNA damage repair through NHEJ, promoting genomic stability, thereby delaying cellular aging and extending organismal lifespan. During genomic stress, SIRT7 helps maintain genomic stability by regulating TP53. Increased SIRT7 expression reduces DNA damage and enhances the expression of cell cycle inhibitors p21/p27, contributing to decreased accumulation of TP53. Moreover, SIRT7 exhibits NAD+ ‐dependent desuccinylase activity, de‐succinylating H3K122, which is vital for maintaining genomic stability [49].

### 3.3 Participation in rDNA Transcription

In addition to chromatin remodeling, histone acetylation levels are critical in gene transcription regulation. The nucleolus is a major site for rRNA synthesis, initial ribosome assembly, and cellular protein processing. Being a nucleolar protein, SIRT7 is closely linked to transcriptional regulation. There are over 100 ribosomal DNA (rDNA) repeat sequences in normal cells, and due to extensive transcription, rDNA is relatively unstable in the genome. Therefore, these repeated sequences of rDNA are easily recombined with each other, leading to the occurrence of diseases. SIRT7 is part of the RNA polymerase I (RNA pol I) complex, interacting with upstream binding factor (UBF) to upregulate rDNA transcription [86]. Chen et al. [73] found that SIRT7 deacetylation of RNA pol I subunit PAF53 affects rDNA transcription, with reduced SIRT7 leading to decreased rRNA precursor synthesis. SIRT7 also regulates RNA pol III expression at the transcriptional level and RNA pol II function at the posttranscriptional level. Proteomic and bioinformatic results further confirm SIRT7's interactions with RNA pol I and UBF, as well as its association with RNA pol II and RNA pol III‐dependent transcription processes and several nucleolar chromatin remodeling complexes [86, 87].

### 3.4 Regulation of Cellular Senescence and Apoptosis

Although the sirtuin protein family was discovered as early as 1979, it garnered scientific attention as a longevity protein. Studies have shown that sirtuins are enzymes that play an important role in aging and prolonging lifespan [88]. Overexpression of Sir2 has been shown to extend yeast lifespan [89]. Similarly, overexpression of sirtuins can prolong the lifespan of nematodes and fruit flies, indicating their involvement in cellular aging and apoptosis regulation [90]. Studies have shown that loss of SIRT7 negatively affects hematopoietic stem cell regeneration potential and phenotypic maintenance. SIRT7 expression was reduced in aged HSCs, and SIRT7 up‐regulation improved the regenerative capacity of aged HSCs [91]. Vakhrusheva et al. [92] found that SIRT7 knockout mice (SIRT7−/−) exhibited signs of premature aging, with both mean and maximum lifespans reduced compared to wild‐type (WT) mice. It is hypothesized that premature aging in SIRT7−/− mice may be related to abnormal rRNA transcription or reduced stem cell adaptability. These findings suggest an association between SIRT7 expression and cellular aging regulation. However, further research is needed to confirm whether SIRT7 overexpression can delay cellular senescence or aging processes.

### 3.5 Tumorigenesis

In addition to being involved in the aging process, SIRT7 is also associated with regulating tumor cell apoptosis and DNA damage response, all processes related to cancer, and plays a key role in chromatin regulation, cell transformation programs, and in vivo tumor formation [48]. As an enzymatically active transcription factor, SIRT7 expression is closely linked to tumor clinicopathological features and patient prognosis, making it a potential target for cancer therapy. Numerous studies have found that SIRT7 is typically highly expressed in various human malignancies, including breast cancer, clear cell renal cell carcinoma, lung adenocarcinoma, prostate adenocarcinoma, hepatocellular carcinoma, and gastric cancer [93, 94, 95, 96].

Lee et al. [97] highlighted the importance of the SIRT7–ELK4 interaction in tumorigenesis. SIRT7 interacts with the ETS transcription factor ELK4, which acts as a transcriptional repressor, deacetylating specific H3K18Ac promoters [98]. This function may explain the elevated SIRT7 expression in cancer and its role in maintaining the transformed phenotype of cancer cells [99, 100, 101]. SIRT7 also mediates cell proliferation by activating the MAPK (Raf–MEK–ERK) signaling pathway, participating in processes such as cell proliferation, differentiation, transformation, metastasis, and apoptosis through G protein‐coupled receptor (GPCR) signaling. Additionally, SIRT7 regulates OC cell proliferation and apoptosis by activating the NF‐κB/P65 signaling pathway. SIRT7 deacetylates FKBP51, promoting protein kinase B (PKB) dephosphorylation and regulating the PI3K–PKB signaling pathway [49]. Myb binding protein 1a (Mybbp1a) is a negative regulator of SIRT7, inhibiting its H3K18Ac deacetylase activity and interacting with various transcription factors to achieve chromatin remodeling and rRNA transcription inhibition [102]. Recent studies have also reported that microRNAs can act as endogenous regulators interacting with SIRT7. In hepatocellular carcinoma (HCC), downregulation of miR‐125a‐5p and miR‐125b leads to increased SIRT7 expression, promoting cancer cell proliferation; similar regulatory pathways have been reported in bladder and colorectal cancer [57]. Higher SIRT7 expression in highly graded breast and PCa tissues correlates with tumor progression and reduced overall patient survival, making SIRT7 a prognostic factor for breast and PCa patients.

## 4 Therapeutic Applications of SIRT7

In recent years, extensive research has revealed the multifaceted roles of SIRT7 and its therapeutic potential in human diseases, making it a promising drug target for cancer therapy. Research showed that SIRT7 mediates the significant upregulation of MCM6 K599 crotonylation (MCM6‐K599cr) under DNA replication stress, primarily due to the disassembly of the MCM2 complex and regulation by RNF8‐mediated ubiquitination. Furthermore, the flavonoid kaempferol has been identified as a modulator of SIRT7, enhancing MCM6 Kcr levels and reducing tumor weight. Notably, its combination with paclitaxel highlights kaempferol as a potential chemotherapeutic target for breast cancer treatment [103]. Similarly, SIRT7 interacts with FOXM1, inhibiting the succinylation of FOXM1 at K259 in HEK‐293 T cells. SH3PXD2A‐AS1 regulates FOXM1 succinylation through SIRT7, promoting cisplatin (DDP) resistance in nonsmall cell lung cancer (NSCLC) cells. This interaction provides a promising therapeutic approach for overcoming chemoresistance in NSCLC [104]. While in pancreatic cancer (PC), SIRT7 regulates GLUT3 expression by binding to its enhancer and modulating H3K122 succinylation, thus influencing gemcitabine sensitivity in PC cells. This discovery unveils a novel mechanism by which SIRT7 affects gemcitabine sensitivity, offering an innovative strategy for clinical combination therapy with gemcitabine in PC [105]. Zinc enhances Parkin acetylation through inhibition of SIRT7 activity, promoting mitochondrial autophagy and inhibiting NLRP3 inflammasome activation and pyroptosis. This mechanism improves outcomes in sepsis‐induced acute kidney injury (SI‐AKI), providing a potential therapeutic avenue for SI‐AKI [106].

In general, SIRT7's involvement in various cellular processes, such as DNA replication, chemoresistance, and mitochondrial function, underscores its therapeutic potential across a range of cancers and diseases. Further exploration of SIRT7‐targeting strategies could lead to innovative treatments for cancer and other pathologies.

## 5 SIRT7 in Prostate Cancer

DNA methylation and histone modifications are two critical epigenetic processes, both playing crucial roles in the onset and progression of PCa [107, 108]. Recent studies have found that SIRT7 plays a vital role in various processes of PCa development, including cancer cell proliferation, differentiation, apoptosis, invasion, and metastasis [96]. In this review, we primarily explore the mechanisms of SIRT7 in PCa and its clinical significance. Given that SIRT7 influences both epigenetics and the malignant phenotype of cancer cells, and considering that epigenetic changes are reversible, SIRT7 presents as an ideal target for cancer therapy.

The dual role of SIRT7 in cancer, acting as both a tumor suppressor and promoter, is primarily determined by its expression levels in tumors, regulation of specific oncogenes and tumor suppressor genes (TSGs), and the tumor microenvironment (TME) [109, 110] However, there is some controversy regarding the expression and function of SIRT7 in PCa. Haider et al. [111] reported that the mRNA expression of SIRT7 remained constant in both normal prostate tissue and precancerous prostate tissue, and that the protein expression levels of SIRT7 were similar in normal prostate epithelial cells and PCa cell lines. In contrast, Barber et al. [112] found that, compared to normal prostate tissue, both the mRNA and protein levels of SIRT7 were significantly overexpressed in human PCa samples. This discrepancy may be attributed to tumor‐specific backgrounds or differences in experimental conditions. Furthermore, SIRT7 expression levels are associated with PCa cell migration but do not affect cell viability. Research has also shown that SIRT7 depletion reduces PC3 cell migration without affecting cancer cell proliferation. Conversely, SIRT7 inhibition has been shown to suppress cell proliferation and induce apoptosis in NIH‐3 T3, HEK‐293, and U‐2OS cells by downregulating RNA pol I [63].

The development and progression of PCa involve the abnormal activation of multiple signaling pathways, including PI3K/Akt, NF‐κB, TGF‐β, and androgen receptor (AR) signaling pathways [113, 114, 115]. Among these, the AR signaling pathway is particularly crucial in PCa, as AR translocates to the nucleus in the presence of testosterone or dihydrotestosterone (DHT), where it regulates the expression of target genes such as PSA, influencing the behavior of PCa cells [116]. A rapid rise in PSA levels is often indicative of AR pathway activation [117] and is associated with poor prognosis [118]. Notably, SIRT7 is significantly upregulated in PCa, with its expression closely linked to both AR and PSA. Depletion of SIRT7 not only suppresses androgen‐mediated autophagy but also regulates the AR signaling pathway by upregulating the SMAD4 protein level. SMAD4, a key regulatory factor in the TGF‐β signaling pathway, shows unchanged mRNA expression in SIRT7‐depleted 22Rv1 cells, but its protein levels increase significantly, accompanied by elevated acetylation of SMAD4 [69]. This suggests that SIRT7 may regulate the stability of SMAD4 through deacetylation, thereby influencing the AR signaling pathway.

Clinically, as a member of the HDACs family, high SIRT7 expression in PCa is detected compared to normal tissues, reflecting the clonality of individual PCa cells and insufficient histone H3 acetylation [119]. Abnormal HDAC activity may disrupt the normal epigenome of prostate cells. HDAC inhibitors (HDACi) have been reported to significantly inhibit androgen receptor (AR) transcriptional activity and suppress PCa cell proliferation [120, 121]. These small molecule inhibitors/drugs can also enhance histone acetylation at specific gene promoter regions, regulating apoptosis and differentiation. Studies show that in androgen‐dependent or independent LNCaP and DU145 cell lines, KHSRP acetylation can be induced by androgen stimulation, but this is not observed in 22RV1 cells. This suggests that the regulatory role of KHSRP acetylation in response to androgen stimulation is complex in different types of PCa cells. One possible reason for this is that the sensitivity to androgen stimulation varies between cell lines due to the presence of either mutant or wild‐type androgen receptors [122, 123]. Interestingly, another bioactive compound, YZL‐51 N, isolated from the extract of *Periplaneta Americana* , can impair SIRT7 enzyme activity by occupying the NAD+ binding pocket, demonstrating its potential in selectively SIRT7 inhibition [124]. Moreover, ERG fusion‐positive PCa cells exhibit abnormal sensitivity to HDACi [125]. SIRT7 overexpression induces resistance to docetaxel (DOC) in PCa cells, suggesting a link between SIRT7 deacetylase activity and chemotherapy resistance. The SIRT7 inhibitor 97,491 exerts its inhibitory effect on SIRT7 in vivo by upregulating caspase‐associated protein, thereby promoting apoptosis and inhibiting tumor growth. It inhibits the deacetylase activity of SIRT7 and prevents tumor progression by enhancing p53 stability through acetylation at the K373/382 sites [126, 127]. In 2019, a study found that two cyclic tripeptides contain sirtuin inhibitory warheads with thiourea and formamide catalytic mechanisms as central residues, which have inhibitory effects on SIRT7 [128]. There are also studies on the synthesis of compounds 2800Z and 40569Z, both of which have strong interactions with the SIRT7 protein and specifically inhibit SIRT7 deacetylation activity in vitro [129]. Additionally, studies have shown that the combined use of the existing chemotherapeutic drugs norcantharidin (NCTD) and paclitaxel (PTX) has therapeutic effects in regulating SIRT7 expression and enhancing anticancer effects [130]. However, more regulatory factors and potential mechanisms in the progression of PCa require further in‐depth research (Table 2).

**TABLE 2:** Regulatory factors and potential mechanisms of SIRT7 in prostate cancer.

Classification	Regulatory genes	Regulatory mechanism
MicroRNA	MiR‐148b	SIRT7 was negatively regulated by miR‐148b through binding to the 3′UTR [131]
MicroRNA	MiR‐770	MiR‐770 binds to 3′‐UTR to inhibit the expression of SIRT7 [3]
MicroRNA	miR‐125a‐5p, miR‐125b	Bioinformatics analysis of target sites and ectopic expression in HCC cells showed that miR‐125a‐5p and miR‐125b suppressed SIRT7 [57]
MicroRNA	miR‐125b‐5p	High glucose induces miR‐125b‐5p, possibly via JAK/STAT, which in turn suppresses SIRT7 expression [132]
Circular RNA	CircPVT1	Downregulation of circular RNA circPVT1 restricts cell growth of hepatocellular carcinoma through downregulation of Sirtuin 7 via microRNA‐3666 [133]
Protein	FBXO7	FBXO7 as a novel E3 ligase for SIRT7 that negatively regulates intracellular SIRT7 levels through SCF‐dependent Lys‐48‐linked polyubiquitination and proteasomal degradation [134]
Protein	HDAC8	HDAC8 as a novel cofactor of SMAD3/4 complex, transcriptionally suppresses *SIRT7* via local chromatin remodeling [135]
Protein	PYCR1	PYCR1‐catalyzed NAD^+^ production stimulates the deacetylation activity of Sirt7 on H3K18ac that restrains genes transcription [136]

## 6 Conclusions and Prospects

In conclusion, extensive research on SIRT7 has revealed its involvement in various biological processes, making it a key regulatory factor for cell survival. SIRT7 plays a crucial role in multiple human diseases, particularly cancer. Generally, SIRT7 is widely considered an oncogene due to its upregulation in many tumor types. However, it may also function as a tumor suppressor gene, especially in advanced cancers, likely due to its tissue‐specific roles. SIRT7's lysine deacetylase activity selectively removes specific histone marks (H3K18Ac), playing a vital role in the epigenetic modifications involved in tumorigenesis. Currently, research on SIRT7 is still in its early stages, with many regulatory mechanisms yet to be understood. Issues such as identifying potential pathways and mechanisms involving SIRT7‐interacting target proteins through proteomics analysis and the dual role of SIRT7 in tumors depending on tissue specificity and different stages of tumorigenesis require further investigation. The role of SIRT7 in cell proliferation and malignant transformation through epigenetic regulation and intracellular balance is not yet clearly elucidated. Moreover, developing more specific inhibitors or activators targeting SIRT7 is essential. Therefore, further research is needed to understand SIRT7's specific roles in regulating cancer progression‐related signaling pathways, enabling its application from molecular biology to clinical practice as a new disease prevention and treatment target, providing new insights for clinical disease prevention and treatment.

## 7 Article Collection and Handling TCGA/cBioPortal Data

### 7.1 Article Collection

- Literature Search: A systematic search was conducted in databases such as PubMed, Google Scholar, and Web of Science, using keywords like “SIRT7,” “cancer therapy,” “TCGA,” and “cBioPortal,” limited to studies published in the last 10 years.
- Inclusion Criteria: Studies that focused on the role of SIRT7 in cancer or its therapeutic potential and utilized TCGA or cBioPortal data for analysis.
- Exclusion Criteria: Excluded non‐English articles, studies with insufficient data, or those not directly related to SIRT7.

### 7.2 Handling TCGA and cBioPortal Data

- Data Sources: TCGA: Data was accessed from the TCGA portal (https://portal.gdc.cancer.gov/) for gene expression, mutation, and clinical data to analyze the relationship between SIRT7 and clinical outcomes. cBioPortal: Data on mutations, amplification, and expression of SIRT7 were extracted from cBioPortal (https://www.cbioportal.org/).
- Data Extraction: RNA‐Seq, mutation data, and other relevant datasets were downloaded for gene expression and survival analysis.
- Data Analysis: Kaplan–Meier survival curves and correlation analyses were used to assess the clinical significance of SIRT7 expression in relation to patient outcomes.

## Author Contributions

**Jiale Zhang:** conceptualization (equal). **Chenxin Liu:** conceptualization (equal). **Wenting Luo:** funding acquisition (equal). **Baoqing Sun:** funding acquisition (equal).

## Ethics Statement

Since TCGA and cBioPortal data are publicly available and de‐identified, no additional ethical approval was required.

## Consent

The authors have nothing to report.

## Conflicts of Interest

The authors declare no conflicts of interest.

## Data Availability

The authors have nothing to report.

## References

[1] S. Imai and L. Guarente , “NAD+ and Sirtuins in Aging and Disease,” Trends in Cell Biology 24, no. 8 (2014): 464–471. 24786309 10.1016/j.tcb.2014.04.002 PMC4112140

[2] T. Finkel , C. X. Deng , and R. Mostoslavsky , “Recent Progress in the Biology and Physiology of Sirtuins,” Nature 460, no. 7255 (2009): 587–591. 19641587 10.1038/nature08197 PMC3727385

[3] H. Li , Z. Yuan , J. Wu , J. Lu , Y. Wang , and L. Zhang , “Unraveling the Multifaceted Role of SIRT7 and Its Therapeutic Potential in Human Diseases,” International Journal of Biological Macromolecules 279, no. Pt 2 (2024): 135210, 10.1016/j.ijbiomac.2024.135210. 39218192

[4] X. Ye , M. Li , T. Hou , T. Gao , W. G. Zhu , and Y. Yang , “Sirtuins in Glucose and Lipid Metabolism,” Oncotarget 8, no. 1 (2017): 1845–1859, 10.18632/oncotarget.12157. 27659520 PMC5352102

[5] N. Poulose and R. Raju , “Sirtuin Regulation in Aging and Injury,” Biochimica et Biophysica Acta 1852, no. 11 (2015): 2442–2455. 26303641 10.1016/j.bbadis.2015.08.017 PMC4682052

[6] V. Carafa , L. Altucci , and A. Nebbioso , “Dual Tumor Suppressor and Tumor Promoter Action of Sirtuins in Determining Malignant Phenotype,” Frontiers in Pharmacology 10 (2019): e38. 10.3389/fphar.2019.00038 PMC6363704 30761005

[7] H. Vaziri , S. K. Dessain , E. Ng Eaton , et al., “hSIR2(SIRT1) Functions as an NAD‐Dependent p53 Deacetylase,” Cell 107, no. 2 (2001): 149–159. 11672523 10.1016/s0092-8674(01)00527-x

[8] M. Tanno , J. Sakamoto , T. Miura , K. Shimamoto , and Y. Horio , “Nucleocytoplasmic Shuttling of the NAD+‐Dependent Histone Deacetylase SIRT1,” Journal of Biological Chemistry 282, no. 9 (2007): 6823–6832, 10.1074/jbc.M609554200. 17197703

[9] J. T. Rodgers , C. Lerin , W. Haas , S. P. Gygi , B. M. Spiegelman , and P. Puigserver , “Nutrient Control of Glucose Homeostasis Through a Complex of PGC‐1alpha and SIRT1,” Nature 434, no. 7029 (2005): 113–118, 10.1038/nature03354. 15744310

[10] X. Li , S. Zhang , G. Blander , J. G. Tse , M. Krieger , and L. Guarente , “SIRT1 Deacetylates and Positively Regulates the Nuclear Receptor LXR,” Molecular Cell 28, no. 1 (2007): 91–106, 10.1016/j.molcel.2007.07.032. 17936707

[11] R. H. Wang , T. J. Lahusen , Q. Chen , et al., “SIRT1 Deacetylates TopBP1 and Modulates Intra‐S‐Phase Checkpoint and DNA Replication Origin Firing,” International Journal of Biological Sciences 10, no. 10 (2014): 1193–1202, 10.7150/ijbs.11066. 25516717 PMC4261203

[12] J. M. Dai , Z. Y. Wang , D. C. Sun , et al., “SIRT1 Interacts With p73 and Suppresses p73‐Dependent Transcriptional Activity,” Journal of Cellular Physiology 210, no. 1 (2007): 161–166. 16998810 10.1002/jcp.20831

[13] Y. Zhang , M. Zhang , H. Dong , et al., “Deacetylation of Cortactin by SIRT1 Promotes Cell Migration,” Oncogene 28, no. 3 (2009): 445–460, 10.1038/onc.2008.388. 18850005

[14] K. Pruitt , R. L. Zinn , J. E. Ohm , et al., “Inhibition of SIRT1 Reactivates Silenced Cancer Genes Without Loss of Promoter DNA Hypermethylation,” PLoS Genetics 2, no. 3 (2006): e40. 16596166 10.1371/journal.pgen.0020040 PMC1420676

[15] A. Noguchi , K. Kikuchi , H. Zheng , et al., “SIRT1 Expression Is Associated With a Poor Prognosis, Whereas DBC1 Is Associated With Favorable Outcomes in Gastric Cancer,” Cancer Medicine 3, no. 6 (2014): 1553–1561, 10.1002/cam4.310. 25146318 PMC4298382

[16] J. Katto , N. Engel , W. Abbas , G. Herbein , and U. Mahlknecht , “Transcription Factor NFκB Regulates the Expression of the Histone Deacetylase SIRT1,” Clinical Epigenetics 5, no. 1 (2013): 11, 10.1186/1868-7083-5-11. 23870485 PMC3727996

[17] L. Bordone , D. Cohen , A. Robinson , et al., “SIRT1 Transgenic Mice Show Phenotypes Resembling Calorie Restriction,” Aging Cell 6, no. 6 (2007): 759–767, 10.1111/j.1474-9726.2007.00335.x. 17877786

[18] Y. Nakahata , M. Kaluzova , B. Grimaldi , et al., “The NAD+‐Dependent Deacetylase SIRT1 Modulates CLOCK‐Mediated Chromatin Remodeling and Circadian Control,” Cell 134, no. 2 (2008): 329–340, 10.1016/j.cell.2008.07.002. 18662547 PMC3526943

[19] G. Asher , D. Gatfield , M. Stratmann , et al., “SIRT1 Regulates Circadian Clock Gene Expression Through PER2 Deacetylation,” Cell 134, no. 2 (2008): 317–328, 10.1016/j.cell.2008.06.050. 18662546

[20] L. Qiao and J. Shao , “SIRT1 Regulates Adiponectin Gene Expression Through Foxo1‐C/Enhancer‐Binding Protein Alpha Transcriptional Complex,” Journal of Biological Chemistry 281, no. 52 (2006): 39915–39924. 17090532 10.1074/jbc.M607215200

[21] S. Perrod , M. M. Cockell , T. Laroche , et al., “A Cytosolic NAD‐Dependent Deacetylase, Hst2p, Can Modulate Nucleolar and Telomeric Silencing in Yeast,” EMBO Journal 20, no. 1–2 (2001): 197–209. 11226170 10.1093/emboj/20.1.197 PMC140183

[22] B. J. North and E. Verdin , “Interphase Nucleo‐Cytoplasmic Shuttling and Localization of SIRT2 During Mitosis,” PLoS One 2, no. 8 (2007): e784. 17726514 10.1371/journal.pone.0000784 PMC1949146

[23] L. Serrano , P. Martínez‐Redondo , A. Marazuela‐Duque , et al., “The Tumor Suppressor SirT2 Regulates Cell Cycle Progression and Genome Stability by Modulating the Mitotic Deposition of H4K20 Methylation,” Genes & Development 27, no. 6 (2013): 639–653, 10.1101/gad.211342.112. 23468428 PMC3613611

[24] B. J. North , M. A. Rosenberg , K. B. Jeganathan , et al., “SIRT2 Induces the Checkpoint Kinase BubR1 to Increase Lifespan,” EMBO Journal 33, no. 13 (2014): 1438–1453. 24825348 10.15252/embj.201386907 PMC4194088

[25] J. Gal , Y. Bang , and H. J. Choi , “SIRT2 Interferes With Autophagy‐Mediated Degradation of Protein Aggregates in Neuronal Cells Under Proteasome Inhibition,” Neurochemistry International 61, no. 7 (2012): 992–1000. 22819792 10.1016/j.neuint.2012.07.010

[26] K. M. Rothgiesser , S. Erener , S. Waibel , et al., “SIRT2 Regulates NF‐κB Dependent Gene Expression Through Deacetylation of p65 Lys310,” Journal of Cell Science 123, no. Pt 24 (2010): 4251–4258. 21081649 10.1242/jcs.073783

[27] B. Schwer , B. J. North , R. A. Frye , M. Ott , and E. Verdin , “The Human Silent Information Regulator (Sir)2 Homologue hSIRT3 Is a Mitochondrial Nicotinamide Adenine Dinucleotide‐Dependent Deacetylase,” Journal of Cell Biology 158, no. 4 (2002): 647–657, 10.1083/jcb.200205057. 12186850 PMC2174009

[28] C. Schlicker , M. Gertz , P. Papatheodorou , B. Kachholz , C. Becker , and C. Steegborn , “Substrates and Regulation Mechanisms for the Human Mitochondrial Sirtuins Sirt3 and Sirt5,” Journal of Molecular Biology 382, no. 3 (2008): 790–801, 10.1016/j.jmb.2008.07.048. 18680753

[29] W. C. Hallows , W. Yu , B. C. Smith , et al., “Sirt3 Promotes the Urea Cycle and Fatty Acid Oxidation During Dietary Restriction,” Molecular Cell 41, no. 2 (2011): 139–149, 10.1016/j.molcel.2011.01.002. 21255725 PMC3101115

[30] M. D. Hirschey , T. Shimazu , E. Goetzman , et al., “SIRT3 Regulates Mitochondrial Fatty‐Acid Oxidation by Reversible Enzyme Deacetylation,” Nature 464, no. 7285 (2010): 121–125, 10.1038/nature08778. 20203611 PMC2841477

[31] H. S. Kim , K. Patel , K. Muldoon‐Jacobs , et al., “SIRT3 Is a Mitochondria‐Localized Tumor Suppressor Required for Maintenance of Mitochondrial Integrity and Metabolism During Stress,” Cancer Cell 17, no. 1 (2010): 41–52, 10.1016/j.ccr.2009.11.023. 20129246 PMC3711519

[32] D. Bellizzi , G. Rose , P. Cavalcante , et al., “A Novel VNTR Enhancer Within the SIRT3 Gene, a Human Homologue of SIR2, Is Associated With Survival at Oldest Ages,” Genomics 85, no. 2 (2005): 258–263, 10.1016/j.ygeno.2004.11.003. 15676284

[33] E. Michishita , J. Y. Park , J. M. Burneskis , J. Barrett , and I. Horikawa , “Evolutionarily Conserved and Nonconserved Cellular Localizations and Functions of Human SIRT Proteins,” Molecular Biology of the Cell 16, no. 10 (2005): 4623–4635, 10.1091/mbc.e05-01-0033. 16079181 PMC1237069

[34] M. C. Haigis , R. Mostoslavsky , K. M. Haigis , et al., “SIRT4 Inhibits Glutamate Dehydrogenase and Opposes the Effects of Calorie Restriction in Pancreatic Beta Cells,” Cell 126, no. 5 (2006): 941–954. 16959573 10.1016/j.cell.2006.06.057

[35] N. Ahuja , B. Schwer , S. Carobbio , et al., “Regulation of Insulin Secretion by SIRT4, a Mitochondrial ADP‐Ribosyltransferase,” Journal of Biological Chemistry 282, no. 46 (2007): 33583–33592. 17715127 10.1074/jbc.M705488200

[36] G. Laurent , V. C. De Boer , L. W. Finley , et al., “SIRT4 Represses Peroxisome Proliferator‐Activated Receptor α Activity to Suppress Hepatic Fat Oxidation,” Molecular and Cellular Biology 33, no. 22 (2013): 4552–4561, 10.1128/MCB.00087-13. 24043310 PMC3838178

[37] M. Miyo , H. Yamamoto , M. Konno , et al., “Tumour‐Suppressive Function of SIRT4 in Human Colorectal Cancer,” British Journal of Cancer 113, no. 3 (2015): 492–499, 10.1038/bjc.2015.226. 26086877 PMC4522635

[38] T. Nakagawa , D. J. Lomb , M. C. Haigis , et al., “SIRT5 Deacetylates Carbamoyl Phosphate Synthetase 1 and Regulates the Urea Cycle,” Cell 137, no. 3 (2009): 560–570. 19410549 10.1016/j.cell.2009.02.026 PMC2698666

[39] Y. Zhang , S. S. Bharathi , M. J. Rardin , et al., “SIRT3 and SIRT5 Regulate the Enzyme Activity and Cardiolipin Binding of Very Long‐Chain Acyl‐CoA Dehydrogenase,” PLoS One 10, no. 3 (2015): e0122297. 25811481 10.1371/journal.pone.0122297 PMC4374878

[40] M. J. Rardin , W. He , Y. Nishida , et al., “SIRT5 Regulates the Mitochondrial Lysine Succinylome and Metabolic Networks,” Cell Metabolism 18, no. 6 (2013): 920–933, 10.1016/j.cmet.2013.11.013. 24315375 PMC4105152

[41] Z. F. Lin , H. B. Xu , J. Y. Wang , et al., “SIRT5 Desuccinylates and Activates SOD1 to Eliminate ROS,” Biochemical and Biophysical Research Communications 441, no. 1 (2013): 191–195. 24140062 10.1016/j.bbrc.2013.10.033

[42] L. Zhou , F. Wang , R. Sun , et al., “SIRT5 Promotes IDH2 Desuccinylation and G6PD Deglutarylation to Enhance Cellular Antioxidant Defense,” EMBO Reports 17, no. 6 (2016): 811–822. 27113762 10.15252/embr.201541643 PMC5278614

[43] G. Liszt , E. Ford , M. Kurtev , and L. Guarente , “Mouse Sir2 Homolog SIRT6 Is a Nuclear ADP‐Ribosyltransferase,” Journal of Biological Chemistry 280, no. 22 (2005): 21313–21320, 10.1074/jbc.M413296200. 15795229

[44] Z. Mao , C. Hine , X. Tian , et al., “SIRT6 Promotes DNA Repair Under Stress by Activating PARP1,” Science (New York, N.Y.) 332, no. 6036 (2011): 1443–1446, 10.1126/science.1202723. 21680843 PMC5472447

[45] H. Jiang , S. Khan , Y. Wang , et al., “SIRT6 Regulates TNF‐α Secretion Through Hydrolysis of Long‐Chain Fatty Acyl Lysine,” Nature 496, no. 7443 (2013): 110–113. 23552949 10.1038/nature12038 PMC3635073

[46] R. Mostoslavsky , K. F. Chua , D. B. Lombard , et al., “Genomic Instability and Aging‐Like Phenotype in the Absence of Mammalian SIRT6,” Cell 124, no. 2 (2006): 315–329. 16439206 10.1016/j.cell.2005.11.044

[47] S. Kiran , N. Chatterjee , S. Singh , S. C. Kaul , R. Wadhwa , and G. Ramakrishna , “Intracellular Distribution of Human SIRT7 and Mapping of the Nuclear/Nucleolar Localization Signal,” FEBS Journal 280, no. 14 (2013): 3451–3466, 10.1111/febs.12346. 23680022

[48] M. F. Barber , E. Michishita‐Kioi , Y. Xi , et al., “SIRT7 Links H3K18 Deacetylation to Maintenance of Oncogenic Transformation,” Nature 487, no. 7405 (2012): 114–118. 22722849 10.1038/nature11043 PMC3412143

[49] L. Li , L. Shi , S. Yang , et al., “SIRT7 Is a Histone Desuccinylase That Functionally Links to Chromatin Compaction and Genome Stability,” Nature Communications 7 (2016): 12235, 10.1038/ncomms12235. PMC4961794 27436229

[50] B. N. Vazquez , J. K. Thackray , N. G. Simonet , et al., “SIRT7 Promotes Genome Integrity and Modulates Non‐Homologous End Joining DNA Repair,” EMBO Journal 35, no. 14 (2016): 1488–1503. 27225932 10.15252/embj.201593499 PMC4884211

[51] A. E. Wyman , Z. Noor , R. Fishelevich , et al., “Sirtuin 7 Is Decreased in Pulmonary Fibrosis and Regulates the Fibrotic Phenotype of Lung Fibroblasts,” American Journal of Physiology Lung Cellular and Molecular Physiology 312, no. 6 (2017): L945–L958. 28385812 10.1152/ajplung.00473.2016 PMC5495944

[52] N. Lee , D. K. Kim , E. S. Kim , et al., “Comparative Interactomes of SIRT6 and SIRT7: Implication of Functional Links to Aging,” Proteomics 14, no. 13–14 (2014): 1610–1622, 10.1002/pmic.201400001. 24782448

[53] M. E. Hubbi , H. Hu , G. D. M. Kshitiz , and G. L. Semenza , “Sirtuin‐7 Inhibits the Activity of Hypoxia‐Inducible Factors,” Journal of Biological Chemistry 288, no. 29 (2013): 20768–20775. 23750001 10.1074/jbc.M113.476903 PMC3774348

[54] D. G. Hardie , “AMP‐Activated/SNF1 Protein Kinases: Conserved Guardians of Cellular Energy,” Nature Reviews Molecular Cell Biology 8, no. 10 (2007): 774–785. 17712357 10.1038/nrm2249

[55] J. Shin , M. He , Y. Liu , et al., “SIRT7 Represses Myc Activity to Suppress ER Stress and Prevent Fatty Liver Disease,” Cell Reports 5, no. 3 (2013): 654–665, 10.1016/j.celrep.2013.10.007. 24210820 PMC3888240

[56] N. R. Sundaresan , S. A. Samant , V. B. Pillai , S. Rajamohan , and M. Gupta , “SIRT3 Is a Stress‐Responsive Deacetylase in Cardiomyocytes That Protects Cells From Stress‐Mediated Cell Death by Deacetylation of Ku70,” Molecular and Cellular Biology 28, no. 20 (2008): 6384–6401, 10.1128/MCB.00426-08. 18710944 PMC2577434

[57] J. K. Kim , J. H. Noh , K. H. Jung , et al., “Sirtuin7 Oncogenic Potential in Human Hepatocellular Carcinoma and Its Regulation by the Tumor Suppressors MiR‐125a‐5p and MiR‐125b,” Hepatology (Baltimore, md) 57, no. 3 (2013): 1055–1067, 10.1002/hep.26101. 23079745

[58] M. F. Blank and I. Grummt , “The Seven Faces of SIRT7,” Transcription 8, no. 2 (2017): 67–74. 28067587 10.1080/21541264.2016.1276658 PMC5423475

[59] V. D. Longo and B. K. Kennedy , “Sirtuins in Aging and Age‐Related Disease,” Cell 126, no. 2 (2006): 257–268. 16873059 10.1016/j.cell.2006.07.002

[60] D. Mcguinness , D. H. Mcguinness , J. A. Mccaul , et al., “Sirtuins, Bioageing, and Cancer,” Journal of Aging Research 2011 (2011): 235754. 21766030 10.4061/2011/235754 PMC3134127

[61] P. T. Schumacker , “A Tumor Suppressor SIRTainty,” Cancer Cell 17, no. 1 (2010): 5–6. 20129243 10.1016/j.ccr.2009.12.032

[62] H. Yamamoto , K. Schoonjans , and J. Auwerx , “Sirtuin Functions in Health and Disease,” Molecular Endocrinology (Baltimore, md) 21, no. 8 (2007): 1745–1755. 17456799 10.1210/me.2007-0079

[63] E. Ford , R. Voit , G. Liszt , et al., “Mammalian Sir2 Homolog SIRT7 Is an Activator of RNA Polymerase I Transcription,” Genes & Development 20, no. 9 (2006): 1075–1080, 10.1101/gad.1399706. 16618798 PMC1472467

[64] S. Kiran , T. Anwar , M. Kiran , and G. Ramakrishna , “Sirtuin 7 in Cell Proliferation, Stress and Disease: Rise of the Seventh Sirtuin!,” Cellular Signalling 27, no. 3 (2015): 673–682, 10.1016/j.cellsig.2014.11.026. 25435428

[65] J. Ford , M. Jiang , and J. Milner , “Cancer‐Specific Functions of SIRT1 Enable Human Epithelial Cancer Cell Growth and Survival,” Cancer Research 65, no. 22 (2005): 10457–10463. 16288037 10.1158/0008-5472.CAN-05-1923

[66] S. Paredes , L. Villanova , and K. F. Chua , “Molecular Pathways: Emerging Roles of Mammalian Sirtuin SIRT7 in Cancer,” Clinical Cancer Research: An Official Journal of the American Association for Cancer Research 20, no. 7 (2014): 1741–1746. 24536059 10.1158/1078-0432.CCR-13-1547 PMC3980586

[67] A. Aljada , A. M. Saleh , M. Alkathiri , et al., “Altered Sirtuin 7 Expression Is Associated With Early Stage Breast Cancer,” Breast Cancer: Basic and Clinical Research 9 (2015): 3–8. 25922576 10.4137/BCBCR.S23156 PMC4396534

[68] X. Tang , L. Shi , N. Xie , et al., “SIRT7 Antagonizes TGF‐β Signaling and Inhibits Breast Cancer Metastasis,” Nature Communications 8, no. 1 (2017): 318, 10.1038/s41467-017-00396-9. PMC5566498 28827661

[69] M. Ding , C. Y. Jiang , Y. Zhang , et al., “SIRT7 Depletion Inhibits Cell Proliferation and Androgen‐Induced Autophagy by Suppressing the AR Signaling in Prostate Cancer,” Journal of Experimental & Clinical Cancer Research 39, no. 1 (2020): 28. 32019578 10.1186/s13046-019-1516-1 PMC6998106

[70] M. Tang , H. Tang , B. Tu , and W. G. Zhu , “SIRT7: A Sentinel of Genome Stability,” Open Biology 11, no. 6 (2021): 210047, 10.1098/rsob.210047. 34129782 PMC8205529

[71] M. Chen , J. Tan , Z. Jin , T. Jiang , J. Wu , and X. Yu , “Research Progress on Sirtuins (SIRTs) Family Modulators,” Biomedicine & Pharmacotherapy = Biomedecine & Pharmacotherapie 174 (2024): 116481, 10.1016/j.biopha.2024.116481. 38522239

[72] S. Voelter‐Mahlknecht , S. Letzel , and U. Mahlknecht , “Fluorescence In Situ Hybridization and Chromosomal Organization of the Human Sirtuin 7 Gene,” International Journal of Oncology 28, no. 4 (2006): 899–908. 16525639

[73] S. Chen , J. Seiler , M. Santiago‐Reichelt , K. Felbel , I. Grummt , and R. Voit , “Repression of RNA Polymerase I Upon Stress Is Caused by Inhibition of RNA‐Dependent Deacetylation of PAF53 by SIRT7,” Molecular Cell 52, no. 3 (2013): 303–313, 10.1016/j.molcel.2013.10.010. 24207024

[74] Z. Tao , Z. Jin , J. Wu , G. Cai , and X. Yu , “Sirtuin Family in Autoimmune Diseases,” Frontiers in Immunology 14 (2023): 1186231, 10.3389/fimmu.2023.1186231. 37483618 PMC10357840

[75] A. Afzaal , K. Rehman , S. Kamal , and M. S. H. Akash , “Versatile Role of Sirtuins in Metabolic Disorders: From Modulation of Mitochondrial Function to Therapeutic Interventions,” Journal of Biochemical and Molecular Toxicology 36, no. 7 (2022): e23047, 10.1002/jbt.23047. 35297126

[76] Q. Geng , H. Peng , F. Chen , et al., “High Expression of Sirt7 Served as a Predictor of Adverse Outcome in Breast Cancer,” International Journal of Clinical and Experimental Pathology 8, no. 2 (2015): 1938–1945. 25973086 PMC4396261

[77] Y. Han , Y. Liu , H. Zhang , et al., “Hsa‐miR‐125b Suppresses Bladder Cancer Development by Down‐Regulating Oncogene SIRT7 and Oncogenic Long Noncoding RNA MALAT1,” FEBS Letters no. 13 (2013): 00780‐1, 10.1016/j.febslet.2013.10.023. 24396870

[78] L. M. Mcglynn , S. Mccluney , N. B. Jamieson , et al., “SIRT3 & SIRT7: Potential Novel Biomarkers for Determining Outcome in Pancreatic Cancer Patients,” PLoS One 10, no. 6 (2015): e0131344, 10.1371/journal.pone.0131344. 26121130 PMC4487247

[79] H. Yu , W. Ye , J. Wu , et al., “Overexpression of sirt7 Exhibits Oncogenic Property and Serves as a Prognostic Factor in Colorectal Cancer,” Clinical Cancer Research: An Official Journal of the American Association for Cancer Research 20, no. 13 (2014): 3434–3445, 10.1158/1078-0432.CCR-13-2952. 24771643

[80] H. L. Wang , R. Q. Lu , S. H. Xie , et al., “SIRT7 Exhibits Oncogenic Potential in Human Ovarian Cancer Cells,” Asian Pacific Journal of Cancer Prevention: APJCP 16, no. 8 (2015): 3573–3577. 25921180 10.7314/apjcp.2015.16.8.3573

[81] M. Nava , P. Dutta , N. R. Zemke , R. Farias‐Eisner , J. V. Vadgama , and Y. Wu , “Transcriptomic and ChIP‐Sequence Interrogation of EGFR Signaling in HER2+ Breast Cancer Cells Reveals a Dynamic Chromatin Landscape and S100 Genes as Targets,” BMC Medical Genomics 12, no. 1 (2019): 32, 10.1186/s12920-019-0477-8. 30736768 PMC6368760

[82] S. Ropero and M. Esteller , “The Role of Histone Deacetylases (HDACs) in Human Cancer,” Molecular Oncology 1, no. 1 (2007): 19–25. 19383284 10.1016/j.molonc.2007.01.001 PMC5543853

[83] E. Seto and M. Yoshida , “Erasers of Histone Acetylation: The Histone Deacetylase Enzymes,” Cold Spring Harbor Perspectives in Biology 6, no. 4 (2014): a018713. 24691964 10.1101/cshperspect.a018713 PMC3970420

[84] L. Li and R. Bhatia , “The Controversial Role of Sirtuins in Tumorigenesis ‐ SIRT7 Joins the Debate,” Cell Research 23, no. 1 (2013): 10–12. 22847743 10.1038/cr.2012.112 PMC3541655

[85] Y. Kim , B. E. Kang , K. Gariani , et al., “Loss of Hepatic Sirt7 Accelerates Diethylnitrosamine (DEN)‐Induced Formation of Hepatocellular Carcinoma by Impairing DNA Damage Repair,” BMB Reports 57, no. 2 (2024): 98–103, 10.5483/BMBRep.2023-0187. 38303560 PMC10910089

[86] A. Grob , P. Roussel , J. E. Wright , B. Mcstay , D. Hernandez‐Verdun , and V. Sirri , “Involvement of SIRT7 in Resumption of rDNA Transcription at the Exit From Mitosis,” Journal of Cell Science 122, no. Pt 4 (2009): 489–498, 10.1242/jcs.042382. 19174463 PMC2714433

[87] Y. C. Tsai , T. M. Greco , and I. M. Cristea , “Sirtuin 7 Plays a Role in Ribosome Biogenesis and Protein Synthesis,” Molecular & Cellular Proteomics: MCP 13, no. 1 (2014): 73–83. 24113281 10.1074/mcp.M113.031377 PMC3879631

[88] F. A. Lagunas‐Rangel , “SIRT7 in the Aging Process,” Cellular and Molecular Life Sciences: CMLS 79, no. 6 (2022): 297. 35585284 10.1007/s00018-022-04342-x PMC9117384

[89] K. T. Howitz , K. J. Bitterman , H. Y. Cohen , et al., “Small Molecule Activators of Sirtuins Extend *Saccharomyces cerevisiae* Lifespan,” Nature 425, no. 6954 (2003): 191–196. 12939617 10.1038/nature01960

[90] L. Perico , G. Remuzzi , and A. Benigni , “Sirtuins in Kidney Health and Disease,” Nature Reviews Nephrology 20, no. 5 (2024): 313–329. 38321168 10.1038/s41581-024-00806-4

[91] M. Mohrin , J. Shin , Y. Liu , et al., “Stem Cell Aging. A Mitochondrial UPR‐Mediated Metabolic Checkpoint Regulates Hematopoietic Stem Cell Aging,” Science (New York, N.Y.) 347, no. 6228 (2015): 1374–1377. 25792330 10.1126/science.aaa2361 PMC4447312

[92] O. Vakhrusheva , C. Smolka , P. Gajawada , et al., “Sirt7 Increases Stress Resistance of Cardiomyocytes and Prevents Apoptosis and Inflammatory Cardiomyopathy in Mice,” Circulation Research 102, no. 6 (2008): 703–710, 10.1161/CIRCRESAHA.107.164558. 18239138

[93] X. Liu , C. Li , Q. Li , H. C. Chang , and Y. C. Tang , “SIRT7 Facilitates CENP‐A Nucleosome Assembly and Suppresses Intestinal Tumorigenesis,” IScience 23, no. 9 (2020): 101461, 10.1016/j.isci.2020.101461. 32861997 PMC7476862

[94] C. Zhang , J. Zhao , J. Zhao , et al., “CYP2E1‐Dependent Upregulation of SIRT7 Is Response to Alcohol Mediated Metastasis in Hepatocellular Carcinoma,” Cancer Gene Therapy 29, no. 12 (2022): 1961–1974, 10.1038/s41417-022-00512-y. 35902730 PMC10832389

[95] F. A. Lagunas‐Rangel , “The Dark Side of SIRT7,” Molecular and Cellular Biochemistry 479, no. 11 (2023): 2843–2861, 10.1007/s11010-023-04869-y. 38064138

[96] M. H. Wu , S. C. Hui , Y. S. Chen , et al., “Norcantharidin Combined With Paclitaxel Induces Endoplasmic Reticulum Stress Mediated Apoptotic Effect in Prostate Cancer Cells by Targeting SIRT7 Expression,” Environmental Toxicology 36, no. 11 (2021): 2206–2216, 10.1002/tox.23334. 34272796

[97] H. S. Lee , W. Jung , E. Lee , et al., “SIRT7, H3K18ac, and ELK4 Immunohistochemical Expression in Hepatocellular Carcinoma,” Journal of Pathology and Translational Medicine 50, no. 5 (2016): 337–344, 10.4132/jptm.2016.05.20. 27498548 PMC5042897

[98] C. K. Galang , W. J. Muller , G. Foos , et al., “Changes in the Expression of Many Ets Family Transcription Factors and of Potential Target Genes in Normal Mammary Tissue and Tumors,” Journal of Biological Chemistry 279, no. 12 (2004): 11281–11292. 14662758 10.1074/jbc.M311887200

[99] N. Ashraf , S. Zino , A. Macintyre , et al., “Altered Sirtuin Expression Is Associated With Node‐Positive Breast Cancer,” British Journal of Cancer 95, no. 8 (2006): 1056–1061, 10.1038/sj.bjc.6603384. 17003781 PMC2360714

[100] F. De Nigris , J. Cerutti , C. Morelli , et al., “Isolation of a SIR‐Like Gene, SIR‐T8, That Is Overexpressed in Thyroid Carcinoma Cell Lines and Tissues,” British Journal of Cancer 86, no. 6 (2002): 917–923, 10.1038/sj.bjc.6600156. 11953824 PMC2364158

[101] R. Frye , “SIRT8 Expressed in Thyroid Cancer Is Actually SIRT7,” British Journal of Cancer 87, no. 12 (2002): 1479. 10.1038/sj.bjc.6600635 PMC2376300 12454781

[102] Y. C. Tsai , T. M. Greco , A. Boonmee , et al., “Functional Proteomics Establishes the Interaction of SIRT7 With Chromatin Remodeling Complexes and Expands Its Role in Regulation of RNA Polymerase I Transcription,” Molecular & Cellular Proteomics: MCP 11, no. 5 (2012): 60–76. 22586326 10.1074/mcp.A111.015156 PMC3418843

[103] H. Song , Z. Guo , K. Xie , et al., “Crotonylation of MCM6 Enhances Chemotherapeutics Sensitivity of Breast Cancer via Inducing DNA Replication Stress,” Cell Proliferation 58 (2024): e13759. 39477811 10.1111/cpr.13759 PMC11839194

[104] Y. Chen , S. Wu , Y. Han , H. Shi , J. Yuan , and W. Cui , “LncRNA SH3PXD2A‐AS1 Facilitates Cisplatin Resistance in Non‐Small Cell Lung Cancer by Regulating FOXM1 Succinylation,” BMC Cancer 24, no. 1 (2024): 848, 10.1186/s12885-024-12624-9. 39020302 PMC11256434

[105] K. Chen , T. Li , H. Diao , et al., “SIRT7 Knockdown Promotes Gemcitabine Sensitivity of Pancreatic Cancer Cell via Upregulation of GLUT3 Expression,” Cancer Letters 598 (2024): 217109, 10.1016/j.canlet.2024.217109. 39002692

[106] J. Guo , Z. Yuan , and R. Wang , “Zn(2+) Improves Sepsis‐Induced Acute Kidney Injury by Upregulating SIRT7‐Mediated Parkin Acetylation,” American Journal of Physiology. Renal Physiology 327, no. 1 (2024): F184–F197. 38779758 10.1152/ajprenal.00337.2023

[107] J. R. Dobosy , J. L. Roberts , V. X. Fu , and D. Jarrard , “The Expanding Role of Epigenetics in the Development, Diagnosis and Treatment of Prostate Cancer and Benign Prostatic Hyperplasia,” Journal of Urology 177, no. 3 (2007): 822–831, 10.1016/j.juro.2006.10.063. 17296351

[108] L. C. Li , P. R. Carroll , and R. Dahiya , “Epigenetic Changes in Prostate Cancer: Implication for Diagnosis and Treatment,” Journal of the National Cancer Institute 97, no. 2 (2005): 103–115. 15657340 10.1093/jnci/dji010

[109] M. Roth and W. Y. Chen , “Sorting out Functions of Sirtuins in Cancer,” Oncogene 33, no. 13 (2014): 1609–1620. 23604120 10.1038/onc.2013.120 PMC4295628

[110] C. X. Deng , “SIRT1, Is It a Tumor Promoter or Tumor Suppressor?,” International Journal of Biological Sciences 5, no. 2 (2009): 147–152. 19173036 10.7150/ijbs.5.147 PMC2631220

[111] R. Haider , F. Massa , L. Kaminski , et al., “Sirtuin 7: A New Marker of Aggressiveness in Prostate Cancer,” Oncotarget 8, no. 44 (2017): 77309–77316, 10.18632/oncotarget.20468. 29100388 PMC5652781

[112] S. Malik , L. Villanova , S. Tanaka , et al., “SIRT7 Inactivation Reverses Metastatic Phenotypes in Epithelial and Mesenchymal Tumors,” Scientific Reports 5 (2015): 9841, 10.1038/srep09841. 25923013 PMC4413894

[113] R. B. Marques , A. Aghai , C. M. A. de Ridder , et al., “High Efficacy of Combination Therapy Using PI3K/AKT Inhibitors With Androgen Deprivation in Prostate Cancer Preclinical Models,” European Urology 67, no. 6 (2015): 1177–1185, 10.1016/j.eururo.2014.08.053. 25220373

[114] M. Malinen , E. A. Niskanen , M. U. Kaikkonen , et al., “Crosstalk Between Androgen and Pro‐Inflammatory Signaling Remodels Androgen Receptor and NF‐kappaB Cistrome to Reprogram the Prostate Cancer Cell Transcriptome,” Nucleic Acids Research 45, no. 2 (2017): 619–630. 27672034 10.1093/nar/gkw855 PMC5314794

[115] P. G. Fournier , P. Juarez , G. Jiang , et al., “The TGF‐Beta Signaling Regulator PMEPA1 Suppresses Prostate Cancer Metastases to Bone,” Cancer Cell 27, no. 6 (2015): 809–821. 25982816 10.1016/j.ccell.2015.04.009 PMC4464909

[116] P. H. Riegman , R. J. Vlietstra , J. A. van der Korput , A. O. Brinkmann , and J. Trapman , “The Promoter of the Prostate‐Specific Antigen Gene Contains a Functional Androgen Responsive Element,” Molecular Endocrinology 5, no. 12 (1991): 1921–1930, 10.1210/mend-5-12-1921. 1724287

[117] G. Attard , J. Richards , and J. S. DE Bono , “New Strategies in Metastatic Prostate Cancer: Targeting the Androgen Receptor Signaling Pathway,” Clinical Cancer Research 17, no. 7 (2011): 1649–1657. 21372223 10.1158/1078-0432.CCR-10-0567 PMC3513706

[118] T. Van den Broeck , R. C. N. van den Bergh , N. Arfi , et al., “Prognostic Value of Biochemical Recurrence Following Treatment With Curative Intent for Prostate Cancer: A Systematic Review,” European Urology 75, no. 6 (2019): 967–987. 30342843 10.1016/j.eururo.2018.10.011

[119] S. Cang , J. Feng , S. Konno , et al., “Deficient Histone Acetylation and Excessive Deacetylase Activity as Epigenomic Marks of Prostate Cancer Cells,” International Journal of Oncology 35, no. 6 (2009): 1417–1422. 19885564 10.3892/ijo_00000459

[120] D. E. Frigo and D. P. McDonnell , “Differential Effects of Prostate Cancer Therapeutics on Neuroendocrine Transdifferentiation,” Molecular Cancer Therapeutics 7, no. 3 (2008): 659–669. 18347151 10.1158/1535-7163.MCT-07-0480

[121] A. Sidana , M. Wang , S. Shabbeer , et al., “Mechanism of Growth Inhibition of Prostate Cancer Xenografts by Valproic Acid,” Journal of Biomedicine & Biotechnology 2012 (2012): 180363. 23093837 10.1155/2012/180363 PMC3471003

[122] F. Alimirah , J. Chen , Z. Basrawala , H. Xin , and D. Choubey , “DU‐145 and PC‐3 Human Prostate Cancer Cell Lines Express Androgen Receptor: Implications for the Androgen Receptor Functions and Regulation,” FEBS Letters 580, no. 9 (2006): 2294–2300, 10.1016/j.febslet.2006.03.041. 16580667

[123] H. Yuan , R. Cai , B. Chen , et al., “Acetylated KHSRP Impairs DNA‐Damage‐Response‐Related mRNA Decay and Facilitates Prostate Cancer Tumorigenesis,” Molecular Oncology 18, no. 9 (2024): 2314–2330, 10.1002/1878-0261.13634. 38501452 PMC11467790

[124] T. S. Kang , Y. M. Yan , Y. Tian , et al., “YZL‐51N Functions as a Selective Inhibitor of SIRT7 by NAD(+) Competition to Impede DNA Damage Repair,” IScience 27, no. 6 (2024): 110014. 38947512 10.1016/j.isci.2024.110014 PMC11214487

[125] M. Björkman , K. Iljin , P. Halonen , et al., “Defining the Molecular Action of HDAC Inhibitors and Synergism With Androgen Deprivation in ERG‐Positive Prostate Cancer,” International Journal of Cancer 123, no. 12 (2008): 2774–2781. 18798265 10.1002/ijc.23885

[126] J. H. Kim , D. Kim , S. J. Cho , et al., “Identification of a Novel SIRT7 Inhibitor as Anticancer Drug Candidate,” Biochemical and Biophysical Research Communications 508, no. 2 (2019): 451–457. 30503501 10.1016/j.bbrc.2018.11.120

[127] H. Shen , X. Qi , Y. Hu , et al., “Targeting Sirtuins for Cancer Therapy: Epigenetics Modifications and Beyond,” Theranostics 14, no. 17 (2024): 6726–6767, 10.7150/thno.100667. 39479446 PMC11519805

[128] S. Li , B. Wu , and W. Zheng , “Cyclic Tripeptide‐Based Potent Human SIRT7 Inhibitors,” Bioorganic & Medicinal Chemistry Letters 29, no. 3 (2019): 461–465. 30578034 10.1016/j.bmcl.2018.12.023

[129] C. Zhang , Y. Li , B. Liu , C. Ning , Y. Wang , and Z. Li , “Discovery of SIRT7 Inhibitor as New Therapeutic Options Against Liver Cancer,” Frontiers in Cell and Developmental Biology 9 (2021): 813233, 10.3389/fcell.2021.813233. 35174171 PMC8841758

[130] O. Guzel , A. Kosem , Y. Aslan , et al., “The Role of Pentraxin‐3, Fetuin‐A and Sirtuin‐7 in the Diagnosis of Prostate Cancer,” Urology Journal 19, no. 3 (2021): 196–201, 10.22037/uj.v18i.6626. 34655076

[131] R. Sun , M. Guo , X. Fan , et al., “MicroRNA‐148b Inhibits the Malignant Biological Behavior of Melanoma by Reducing Sirtuin 7 Expression Levels,” BioMed Research International 2020 (2020): 9568976, 10.1155/2020/9568976. 33274232 PMC7700026

[132] S. Vigili de Kreutzenberg , A. Giannella , G. Ceolotto , et al., “A miR‐125/Sirtuin‐7 Pathway Drives the Pro‐Calcific Potential of Myeloid Cells in Diabetic Vascular Disease,” Diabetologia 65, no. 9 (2022): 1555–1568, 10.1007/s00125-022-05733-2. 35708762 PMC9345831

[133] Y. Li , H. Shi , J. Yuan , et al., “Downregulation of Circular RNA circPVT1 Restricts Cell Growth of Hepatocellular Carcinoma Through Downregulation of Sirtuin 7 via microRNA‐3666,” Clinical and Experimental Pharmacology & Physiology 47, no. 7 (2020): 1291–1300. 32017171 10.1111/1440-1681.13273

[134] S. H. Lee , Y. J. Lee , S. Jung , et al., “E3 Ligase Adaptor FBXO7 Contributes to Ubiquitination and Proteasomal Degradation of SIRT7 and Promotes Cell Death in Response to Hydrogen Peroxide,” Journal of Biological Chemistry 299, no. 3 (2023): 102909. 36646384 10.1016/j.jbc.2023.102909 PMC9971319

[135] X. Tang , G. Li , F. Su , et al., “HDAC8 Cooperates With SMAD3/4 Complex to Suppress SIRT7 and Promote Cell Survival and Migration,” Nucleic Acids Research 48, no. 6 (2020): 2912–2923, 10.1093/nar/gkaa039. 31970414 PMC7102950

[136] K. Zheng , N. Sha , G. Hou , et al., “IGF1R‐Phosphorylated PYCR1 Facilitates ELK4 Transcriptional Activity and Sustains Tumor Growth Under Hypoxia,” Nature Communications 14, no. 1 (2023): 6117, 10.1038/s41467-023-41658-z. PMC10542766 37777542

